# Supplementary material for: Occupational Exposure to Halogenated Anaesthetic Gases in Hospitals: A Systematic Review of Methods and Techniques to Assess Air Concentration Levels
Source: Int J Environ Res Public Health. 2022 Dec 28;20(1):514. doi: 10.3390/ijerph20010514 (PMC9819674; doi:10.3390/ijerph20010514)
Supplement: Supplementary file 1 [file ijerph-20-00514-s001.zip › ijerph-2091067-supplementary.pdf]

Review

Occupational exposure to halogenated anaesthetic gases in hospitals: a systematic review of methods and techniques to assess air concentration levels

Marta Keller [1], Andrea Cattaneo[1*], Andrea Spinazzè[1], Letizia Carrozzo[1], Davide Campagnolo[1], Sabrina Rovelli[1], Francesca Borghi[1], Giacomo Fanti[1], Silvia Fustinoni[2,3], Mariella Carrieri[4], Angelo Moretto[4], Domenico Maria Cavallo[1]

[1] Department of Science and High Technology, University of Insubria, Como, Italy
[2] IRCCS Ca' Granda Foundation Maggiore Policlinico Hospital, Milan, Italy
[3] Department of Clinical Sciences and Community Health, University of Milan, Milan, Italy
[4] Department of Cardiac, Thoracic, Vascular Sciences and Public Health, University of Padua, Padua, Italy

* Correspondence: andrea.cattaneo@uninsubria.it

SUPPLEMENTARY MATERIALS

Table S1 Summary of limit values for the halogenated anaesthetic gases isoflurane, desflurane and sevoflurane in the workplace recovered from (IFA, 2015).
(1) mean value 15 minutes

	**Isoflurane $C_3H_2ClF_5O$ CAS 26675-46-7**				**Sevoflurane $C_4H_3F_7O$ CAS 28523-86-6**				**Desflurane $C_3H_2F_6O$ CAS 57041-67-5**			
	Limit value – 8 h		Limit value – Short term		Limit value – 8 h		Limit value – Short term		Limit value – 8 h		Limit value – Short term	
	ppm	mg/m^3	ppm	mg/m^3	ppm	mg/m^3	ppm	mg/m^3	ppm	mg/m^3	ppm	mg/m^3
**Austria**	10	80	20	160								
**Canada-Ontario**	2	15										
**Denmark**	5	38	10 (1)	76 (1)					5	35	10 (1)	70 (1)
**Finland**	10	77	20 (1)	150 (1)	10	83	20 (1)	170 (1)	10	70	20 (1)	140 (1)
**Ireland**	50	380										
**Israel**	2	15	6	45 (1)			2 (1)	16 (1)				
**Norway**	2	15			5	35			5	35		
**Poland**		32				55				125		
**Spain**	50	383										
**Sweden**	10	80	20 (1)	150 (1)	10	80	20 (1)	170 (1)	10	70	20 (1)	140 (1)
**Switzerland**	10	77	80	616								
**UK**	50	383										

Table S2 Articles considered in this systematic review

Reference	Title
(Ji et al., 2021)	Effects of sevoflurane exposure on apoptosis and cell cycle of peripheral blood lymphocytes, and immunologic function
(Hua et al., 2021)	Effects of Occupational Exposure to Waste Anesthetic Gas on Oxidative Stress and DNA Damage
(Dehghani et al., 2021)	Probabilistic health risk assessment of occupational exposure to isoflurane and sevoflurane in the operating room
(Norton et al., 2020)	Assessment of anesthetic gases in a central hospital
(Neghab et al., 2020)	Association between genotoxic properties of inhalation anesthetics and oxidative stress biomarkers
(Jafari et al., 2020)	Effects of occupational exposure to trace levels of halogenated anesthetics on the liver, kidney, and oxidative stress parameters in operating room personnel
(Braz et al., 2020)	High concentrations of waste anesthetic gases induce genetic damage and inflammation in physicians exposed for three years: A cross-sectional study
(Herzog-Niescery et al., 2020)	Comparison of 3 Methods to Assess Occupational Sevoflurane Exposure in Abdominal Surgeons: A Single-Center Observational Pilot Study
(Herzog-Niescery, Vogelsang, Bellgardt, et al., 2019)	The Personnel's Sevoflurane Exposure in the Postanesthesia Care Unit Measured by Photoacoustic Gas Monitoring and Hexafluoroisopropanol Biomonitoring

(Cheng et al., 2019)	Pilot studies of VOC exposure profiles during surgical operations
(Cakmak et al., 2019)	Genetic damage of operating and recovery room personnel occupationally exposed to waste anaesthetic gases
(Herzog-Niescery, Vogelsang, Gude, et al., 2019)	Environmental safety: Air pollution while using MIRUS™ for short-term sedation in the ICU
(Williams et al., 2019)	Evaluation and control of waste anesthetic gas in the postanesthesia care unit within patient and caregiver breathing zones
(Kampan, 2019)	Air quality and employee hygiene-related behavior in a post anesthesia care unit in Thailand
(Amiri et al., 2018)	Early, subclinical hematological changes associated with occupational exposure to high levels of nitrous oxide
(Heiderich et al., 2018)	Low anaesthetic waste gas concentrations in postanaesthesia care unit
(Jafari et al., 2018)	Environmental and biological measurements of isoflurane and sevoflurane in operating room personnel
(Herzog-Niescery et al., 2018)	The impact of the anesthetic conserving device on occupational exposure to isoflurane among intensive care healthcare professionals
(Jan-Peter Özelsel et al., 2018)	Elevated waste anaesthetic gas concentration in the paediatric postanaesthesia care unit
(Herzog-Niescery et al., 2017)	The child's behavior during inhalational induction and its impact on the anesthesiologist's sevoflurane exposure
(Gobbo Braz et al., 2017)	Comparison of waste anesthetic gases in operating rooms with or without an scavenging system in a Brazilian University Hospital
(Souza et al., 2016)	Occupational exposure to anesthetics leads to genomic instability, cytotoxicity and proliferative changes
(Cheung et al., 2016)	Postoperative environmental anesthetic vapour concentrations following removal of the airway device in the operating room versus the postanesthesia care unit

(Herzog-Niescery et al., 2016)	Surgeons' exposure to sevoflurane during paediatric adenoidectomy: A comparison of three airway devices
(Sárkány et al., 2016)	Does standing or sitting position of the anesthesiologist in the operating theatre influence sevoflurane exposure during craniotomies?
(Herzog-Niescery et al., 2015)	Occupational chronic sevoflurane exposure in the everyday reality of the anesthesia workplace
(Ghimenti et al., 2015)	Determination of sevoflurane and isopropyl alcohol in exhaled breath by thermal desorption gas chromatography-mass spectrometry for exposure assessment of hospital staff
(Kunze et al., 2015)	Multi-capillary column-ion mobility spectrometry (MCC-IMS) as a new method for the quantification of occupational exposure to sevoflurane in anaesthesia workplaces: An observational feasibility study
(Jankowska et al., 2015)	Application of predictive models for estimation of health care workers exposure to sevoflurane
(Hiller et al., 2015)	Evaluation of Waste Anesthetic Gas in the Postanesthesia Care Unit within the Patient Breathing Zone
(Chaoul et al., 2015)	Does occupational exposure to anesthetic gases lead to increase of pro-inflammatory cytokines?
(Scapellato et al., 2014)	Biomonitoring occupational sevoflurane exposure at low levels by urinary sevoflurane and hexafluoroisopropanol
(González-Rodríguez et al., 2014)	Health worker exposure risk during inhalation sedation with sevoflurane using the (AnaConDa®) anaesthetic conserving device
(Mcglothlin et al., 2014)	Evaluation and control of waste anesthetic gases in the postanesthesia care unit

(Gianella et al., 2014)	Quantitative chemical analysis of surgical smoke generated during laparoscopic surgery with a vessel-sealing device
(Zare Sakhvidi et al., 2013)	Application of mathematical models in combination with monte carlo simulation for prediction of isoflurane concentration in an operation room theater
(Pickworth et al., 2013)	The scavenging of volatile anesthetic agents in the cardiovascular intensive care unit environment: A technical report
(Zaffina et al., 2012)	Occupational exposure to sevoflurane in pediatric operating rooms: The multi-point sampling method for risk assessment
(Blokker-Veldhuis et al., 2011)	Occupational exposure to sevoflurane during cardiopulmonary bypass
(Heijbel et al., 2010)	Personnel breathing zone sevoflurane concentration adherence to occupational exposure limits in conjunction with filling of vaporisers
(Schebesta et al., 2010)	Exposure to anaesthetic trace gases during general anaesthesia: CobraPLA vs. LMA classic
(Rahe-Meyer et al., 2009)	Comparison of breathing tube connectors during invasive bronchial procedures
(Tankó et al., 2009)	The relative exposure of the operating room staff to sevoflurane during intracerebral surgery
(Al-Ghanem et al., 2008)	Monitoring of volatile anesthetics in operating room personnel using GC-MS
(Ritzu et al., 2007)	Anesthetic gases exposure: Findings from a 13 year environmental and biological monitoring in a hospital company
(Barberio et al., 2006)	Pollution of ambient air by volatile anesthetics: A comparison of 4 anesthetic management techniques
(Eroglu et al., 2006)	A comparison of sister chromatid exchanges in lymphocytes of anesthesiologists to nonanesthesiologist in the same hospital

(Accorsi et al., 2005)	Urinary sevoflurane and hexafluoro-isopropanol as biomarkers of low-level occupational exposure to sevoflurane
(Sackey et al., 2005)	Ambient isoflurane pollution and isoflurane consumption during intensive care unit sedation with the Anesthetic Conserving Device
(Rovesti et al., 2005)	Monitoring occupational exposure to volatile anaesthetics in the operating theatre: environmental and biological measurements
(Ansalone et al., 2004)	Biological monitoring of occupational exposure to low sevoflurane concentrations: A new technique for measuring urinary sevoflurane
(Zanetti et al., 2004)	Longitudinal study (16 years) of the reproductive health of 61 female workers exposed to known levels of volatile anaesthetics
(Gentili et al., 2004)	Exposure of personnel to sevoflurane during paediatric anaesthesia: Influence of professional role and anaesthetic procedure
(Li et al., 2004)	A local scavenging system to remove waste anesthetic gases during general anesthesia
(Summer et al., 2003)	Sevoflurane in exhaled air of operating room personnel
(Raj et al., 2003)	Evaluation of personal, environmental and biological exposure of paediatric anaesthetists to nitrous oxide and sevoflurane
(Proietti et al., 2003)	Anaesthesia techniques, occupational exposure and early neurobehavioral effect
(Mierdl et al., 2003)	Occupational exposure to inhalational anesthetics during cardiac surgery on cardiopulmonary bypass
(Alessio et al., 2003)	Biological monitoring of occupational exposure to Desflurane
(Accorsi et al., 2003)	Proposal for single and mixture biological exposure limits for sevoflurane and nitrous oxide at low occupational exposure levels

(Barbic et al., 2003)	Urinary hexafluoroisopropanol in the evaluation of occupational exposure to sevoflurane: Methodological aspects and critical points
(Cope et al., 2002)	Phase II collaborative pilot study: Preliminary analysis of central neural effects from exposure to volatile anesthetics in the PACU
(Li et al., 2002)	Personnel exposure to waste sevoflurane and nitrous oxide during general anesthesia with cuffed endotracheal tube
(Rieder et al., 2002)	Monitoring pollution by proton-transfer-reaction mass spectrometry during paediatric anaesthesia with positive pressure ventilation via the laryngeal mask airway or uncuffed tracheal tube
(Tanser & Johnson, 2002)	Evaluation of a new paediatric scavenging valve
(Virgili et al., 2002)	Occupational exposure to anesthetic gases at several hospitals
(Gustorff et al., 2002)	Environmental monitoring of sevoflurane and nitrous oxide using the cuffed oropharyngeal airway
(Imbriani et al., 2001)	Biological monitoring of occupational exposure to sevoflurane
(Rieder et al., 2001)	Online monitoring of air quality at the postanesthetic care unit by proton-transfer-reaction mass spectrometry
(Byhahn et al., 2001)	Surgeon's occupational exposure to nitrous oxide and sevoflurane during pediatric surgery
(Hoerauf et al., 2001)	Waste gas exposure to sevoflurane and nitrous oxide during anaesthesia using the oesophageal-tracheal Combitube small adult
(Wiesner et al., 2001)	A follow-up study on occupational exposure to inhaled anaesthetics in Eastern European surgeons and circulating nurses
(Henderson & Matthews, 2000)	Staff exposure to anaesthetic gases in theatre and non theatre areas
(Haufroid et al., 2000)	Biological monitoring of exposure to sevoflurane in operating room personnel by the measurement of hexafluoroisopropanol and fluoride in urine
(Sitarek et al., 2000)	Concentrations of anaesthetic gases in hospital operating theatres

(Byhahn et al., 2000)	Occupational exposure to nitrous oxide and desflurane during ear-nose-throat-surgery
(Proietti et al., 2000)	Environmental monitoring and health monitoring for personnel exposed to inhalation anesthetics
(Wiesner et al., 2000)	Occupational exposure to inhaled anaesthetics: A follow-up study on anaesthetists of eastern European university hospital
(Henderson & Matthews, 1999)	An environmental survey of compliance with occupational exposure standards (OES) for anaesthetic gases
(Hoerauf, Hartmann, et al., 1999)	Occupational exposure to sevoflurane during sedation of adult patients
(Hoerauf, Wallner, et al., 1999)	Exposure to sevoflurane and nitrous oxide during four different methods of anesthetic induction
(Sessler & Badgwell, 1998)	Exposure of postoperative nurses to exhaled anesthetic gases
(Hobbhahn et al., 1998)	Waste gas exposure during desflurane and isoflurane anaesthesia
(Imbriani et al., 1998)	The biological monitoring of inhalation anaesthetics.
(Lucchini et al., 1995)	Neurobehavioral functions in operating theatre personnel: a multicenter study
(Prado et al., 1997)	Biological monitoring of occupational exposure to isoflurane by measurement of isoflurane exhaled breath
(Hoerauf, Harth, et al., 1997)	Occupational exposure to sevoflurane, halothane and nitrous oxide during paediatric anaesthesia. Waste gas exposure during paediatric anaesthesia
(Hoerauf, Funk, et al., 1997)	Occupational exposure to desflurane and isoflurane during cardiopulmonary bypass: Is the gas outlet of the membrane oxygenator an operating theatre pollution hazard?
(Hall et al., 1997)	Environmental monitoring during gaseous induction with sevoflurane

(Hoerauf, Koller, Taeger, et al., 1996)	Occupational exposure to sevoflurane and nitrous oxide in operating room personnel
(Hoerauf, Koller, Jakob, et al., 1996)	Isoflurane waste gas exposure during general anaesthesia: The laryngeal mask compared with tracheal intubation
(De-Amici et al., 1996)	Qualitative characteristics of human exposure to air chemical pollutants in operating rooms
(Domenegati et al., 1996)	Evaluation of a protocol of health surveillance for the personnel exposed to inhalation anesthetics in a sample of 3 operating rooms
(Imberti et al., 1995)	Low flow anaesthesia reduces occupational exposure to inhalation anaesthetics Environmental and biological measurements in operating room personnel
(Imbriani et al., 1995)	Anesthetic in urine as biological index of exposure in operating-room personnel
(Coleman et al., 1994)	Prevention of atmospheric contamination during isoflurane sedation
(Buratti et al., 1993)	The biological monitoring of occupational exposure to anesthetic gas and vapors: the determination of nitrogen protoxide, halothane and isoflurane in the urine
(Marracini et al., 1992)	Evaluation of several neuropsychological parameters in subjects occupationally exposed to anesthetics
(Dang et al., 1992)	Theatre staff members and exposure to halogenated agents
(Imbriani & Ghittori, 1988)	Evaluation of exposure to isoflurane (Forane): Environmental and biological measurements in operating room personnel
(Ravagli et al., 1987)	Anesthetics vapors and gases and health hazards: results of an environmental survey

Table S3 Anaesthetic gases considered in the studies under review. Mixed collects articles with more than one anaesthetic gas.

Anaesthetic gas	Number of papers		Reference
Sevoflurane	49	90s= 5<br>2000s= 20<br>2010s= 24	(Accorsi et al., 2003, 2005; Ansalone et al., 2004; Barbic et al., 2003; Blokker-Veldhuis et al., 2011; Byhahn et al., 2001; Cakmak et al., 2019; Cheng et al., 2019; Eroglu et al., 2006; Gentili et al., 2004; Ghimenti et al., 2015; Gianella et al., 2014; González-Rodríguez et al., 2014; Gustorff et al., 2002; Hall et al., 1997; Haufroid et al., 2000; Heiderich et al., 2018; Heijbel et al., 2010; Henderson & Matthews, 2000; Herzog-Niescery et al., 2015, 2016, 2017, 2020; Herzog-Niescery, Vogelsang, Bellgardt, et al., 2019; Hiller et al., 2015; Hoerauf et al., 2001; Hoerauf, Harth, et al., 1997; Hoerauf, Hartmann, et al., 1999; Hoerauf, Koller, Taeger, et al., 1996; Hoerauf, Wallner, et al., 1999; Hua et al., 2021; Imbriani et al., 2001; Jankowska et al., 2015; Ji et al., 2021; Kunze et al., 2015; Li et al., 2002, 2004; Mcglothlin et al., 2014; Özelsel et al., 2018; Rahe-Meyer et al., 2009; Raj et al., 2003; Rieder et al., 2002; Sárkány et al., 2016; Scapellato et al., 2014; Schebesta et al., 2010; Summer et al., 2003; Tankó et al., 2009; Zaffina et al., 2012; Zanetti et al., 2004)
Isoflurane	26	90s= 15<br>2000s= 9<br>2010s= 2	(Buratti et al., 1993; Coleman et al., 1994; Cope et al., 2002; Dang et al., 1992; De-Amici et al., 1996; Domenegati et al., 1996; Henderson & Matthews, 1999; Herzog-Niescery et al., 2018; Hoerauf, Koller, Jakob, et al., 1996; Imberti et al., 1995; Imbriani et al., 1995, 1998; Imbriani & Ghittori, 1988; Lucchini et al., 1995; Marracini et al., 1992; Prado et al., 1997; Proietti et al., 2000, 2003; Ravagli et al., 1987; Rovesti et al., 2005; Sackey et al., 2005; Sitarek et al., 2000; Tanser & Johnson, 2002; Wiesner et al., 2000, 2001; Zare Sakhvidi et al., 2013)
Desflurane	2	2000s= 1<br>2010s= 1	(Kampan 2019; Byhahn et al. 2000)
Mixed	24	90s= 3<br>2000s= 7<br>2010s= 14	(Al-Ghanem et al., 2008; Alessio et al., 2003; Amiri et al., 2018; Barberio et al., 2006; Braz et al., 2020; Chaoul et al., 2015; Cheung et al., 2016; Dehghani et al., 2021; Gobbo Braz et al., 2017; Herzog-Niescery, Vogelsang, Gude, et al., 2019; Hobbhahn et al., 1998; Hoerauf, Harth, et al., 1997; Jafari et al., 2018, 2020; Mierdl et al., 2003; Neghab et al., 2020; Norton et al., 2020; Pickworth et al., 2013; Rieder et al., 2001; Ritzu et al., 2007; Sessler & Badgwell, 1998; Souza et al., 2016; Virgili et al., 2002; Williams et al., 2019)

Table S4 Hospital environments present in the studies under review. Mixed collects articles with more than one environment.

Hospital environments	Number of papers	Reference
Operating rooms	77	(Accorsi et al., 2003, 2005; Al-Ghanem et al., 2008; Alessio et al., 2003; Amiri et al., 2018; Ansalone et al., 2004; Barberio et al., 2006; Barbic et al., 2003; Blokker-Veldhuis et al., 2011; Braz et al., 2020; Buratti et al., 1993; Byhahn et al., 2000, 2001; Chaoul et al., 2015; Cheng et al., 2019; Dang et al., 1992; De-Amici et al., 1996; Dehghani et al., 2021; Domenegati et al., 1996; Eroglu et al., 2006; Gentili et al., 2004; Ghimenti et al., 2015; Gianella et al., 2014; Gobbo Braz et al., 2017; Gustorff et al., 2002; Haufroid et al., 2000; Heijbel et al., 2010; Herzog-Niescery et al., 2015, 2016, 2017, 2020; Hobbhahn et al., 1998; Hoerauf et al., 2001; Hoerauf, Funk, et al., 1997; Hoerauf, Harth, et al., 1997; Hoerauf, Koller, Jakob, et al., 1996; Hoerauf, Koller, Taeger, et al., 1996; Hoerauf, Wallner, et al., 1999; Hua et al., 2021; Imberti et al., 1995; Imbriani et al., 1995, 1998, 2001; Imbriani & Ghittori, 1988; Jafari et al., 2018, 2020; Jankowska et al., 2015; Ji et al., 2021; Li et al., 2002, 2004; Lucchini et al., 1997; Marracini et al., 1992; Mierdl et al., 2003; Neghab et al., 2020; Prado et al., 1997; Proietti et al., 2000, 2003; Rahe-Meyer et al., 2009; Raj et al., 2003; Ravagli et al., 1987; Rieder et al., 2002; Ritzu et al., 2007; Rovesti et al., 2005; Sárkány et al., 2016; Scapellato et al., 2014; Schebesta et al., 2010; Sitarek et al., 2000; Souza et al., 2016; Summer et al., 2003; Tankó et al., 2009; Virgili et al., 2002; Wiesner et al., 2000, 2001; Zaffina et al., 2012; Zanetti et al., 2004; Zare Sakhvidi et al., 2013)
Postanesthesia care units	15	(Cope et al., 2002; González-Rodríguez et al., 2014; Heiderich et al., 2018; Henderson & Matthews, 1999; Herzog-Niescery, Vogelsang, Bellgardt, et al., 2019; Hiller et al., 2015; Kampan, 2019; Kunze et al., 2015; Mcglothlin et al., 2014; Norton et al., 2020; Özelsel et al., 2018; Rieder et al., 2001; Sackey et al., 2005; Sessler & Badgwell, 1998; Williams et al., 2019)
Intensive care units	4	(Coleman et al., 1994; Herzog-Niescery et al., 2018; Herzog-Niescery, Vogelsang, Gude, et al., 2019; Pickworth et al., 2013)
Anaesthesia rooms	2	(Tanser and Johnson 2002; Hall et al. 1997)
Mixed	3	(Cakmak et al., 2019; Cheung et al., 2016; Henderson & Matthews, 2000)

Table S5 Techniques considered in the studies under review. Mixed collects articles with real-time and time-integrated techniques.

Technique	Number of papers	Reference
Real-time	57	(Ansalone et al., 2004; Barberio et al., 2006; Blokker-Veldhuis et al., 2011; Braz et al., 2020; Buratti et al., 1993; Byhahn et al., 2000, 2001; Chaoul et al., 2015; Cheung et al., 2016; Coleman et al., 1994; Domenegati et al., 1996; Eroglu et al., 2006; Gianella et al., 2014; Gobbo Braz et al., 2017; Gustorff et al., 2002; Hall et al., 1997; Heiderich et al., 2018; Heijbel et al., 2010; Henderson & Matthews, 2000; Herzog-Niescery et al., 2015, 2016, 2017, 2018, 2020; Herzog-Niescery, Vogelsang, Bellgardt, et al., 2019; Hiller et al., 2015; Hobbhahn et al., 1998; Hoerauf, Funk, et al., 1997; Hoerauf, Harth, et al., 1997; Hoerauf, Hartmann, et al., 1999; Hoerauf, Koller, Jakob, et al., 1996; Hoerauf, Koller, Taeger, et al., 1996; Hoerauf, Wallner, et al., 1999; Kampan, 2019; Kunze et al., 2015; Li et al., 2002, 2004; Lucchini et al., 1997; Mcglothlin et al., 2014; Mierdl et al., 2003; Norton et al., 2020; Özelsel et al., 2018; Pickworth et al., 2013; Proietti et al., 2000, 2003; Rahe-Meyer et al., 2009; Rieder et al., 2001, 2002; Rovesti et al., 2005; Schebesta et al., 2010; Sitarek et al., 2000; Souza et al., 2016; Summer et al., 2003; Tanser & Johnson, 2002; Wiesner et al., 2000, 2001; Williams et al., 2019; Zaffina et al., 2012)
Time-integrated	41	(Accorsi et al., 2005; Al-Ghanem et al., 2008; Alessio et al., 2003; Amiri et al., 2018; Barbic et al., 2003; Cakmak et al., 2019; Cheng et al., 2019; Coleman et al., 1994; Cope et al., 2002; Dang et al., 1992; De-Amici et al., 1996; Dehghani et al., 2021; Gentili et al., 2004; Ghimenti et al., 2015; González-Rodríguez et al., 2014; Haufroid et al., 2000; Hua et al., 2021; Imberti et al., 1995; Imbriani et al., 1995, 1998, 2001; Imbriani & Ghittori, 1988; Jafari et al., 2018, 2020; Jankowska et al., 2015; Ji et al., 2021; Kampan, 2019; Marracini et al., 1992; Neghab et al., 2020; Prado et al., 1997; Raj et al., 2003; Ravagli et al., 1987; Rieder et al., 2001; Sackey et al., 2005; Sárkány et al., 2016; Scapellato et al., 2014; Sessler & Badgwell, 1998; Sitarek et al., 2000; Summer et al., 2003; Tankó et al., 2009; Zare Sakhvidi et al., 2013)
Mixed	3	(Henderson & Matthews, 1999; Ritzu et al., 2007; Virgili et al., 2002)

Table S6 Real-time samplers for anaesthetic gases present in the articles in question

[1]PAS= photoacoustic spectroscopy; IR= infrared spectrophotometry; FT-IR= Fourier transform infrared spectroscopy IMS= ion mobility spectrometer; PTR-MS= proton-transfer-reaction mass spectrometry. n.a. = not available

**Analytic System**	**Dimension (cm)** ***Weight (Kg)***	**Detection range** ***Resolution***	**Sampling frequency (s)**	**Interferences**	**References**
PAS[1]	17.5 x 39.5 x 30.0 *9*	0.01 *ppm*	60	N_2O and alcohol	(Ansalone et al., 2004; Blokker-Veldhuis et al., 2011; Byhahn et al., 2000, 2001; Domenegati et al., 1996; Gustorff et al., 2002; Herzog-Niescery et al., 2020; Herzog-Niescery, Vogelsang, Bellgardt, et al., 2019; Herzog-Niescery, Vogelsang, Gude, et al., 2019; Herzog-Niescery et al., 2015, 2016, 2017, 2018; Hobbhahn et al., 1998; Hoerauf, Funk, et al., 1997; Hoerauf, Harth, et al., 1997; Hoerauf, Hartmann, et al., 1999; Hoerauf, Koller, Jakob, et al., 1996; Hoerauf, Koller, Taeger, et al., 1996; Hoerauf, Wallner, et al., 1999; Lucchini et al., 1997; Mierdl et al., 2003; Proietti et al., 2000, 2003; Rahe-Meyer et al., 2009; Rovesti et al., 2005; Schebesta et al., 2010; Wiesner et al., 2000, 2001; Zaffina et al., 2012)
IR[1]	38.1 x 36.5 x 19.1 *8.2*	0.01-0.2 *ppm*	20	CO_2 and water vapor	(Norton et al. 2020; Braz et al. 2020; Williams et al. 2019; Özelsel et al. 2018; Gobbo Braz et al. 2017; Souza et al. 2016; Cheung et al. 2016; Chaoul et al. 2015; Hiller et al. 2015; Mcglothlin, Moenning, and Cole 2014; Pickworth et al. 2013; Heijbel, BjurstÖm, and Jakobsson 2010; Joy Barberio et al. 2006; Li et al. 2002; Tanser and Johnson 2002; K. H. Hoerauf et al. 2001; Henderson and Matthews 2000; Hall et al. 1997; Coleman et al. 1994; Buratti et al. 1993)

FT-IR[1]	45 x 30 x 21<br>*13*	n.a.	100 spectra/sec	CO_2 and water vapor	(Eroglu et al., 2006; Gianella et al., 2014; Li et al., 2002)
IMS[1]	n.a.<br>*15*	*ppt*	n.a.	n.a.	(Heiderich et al., 2018; Kunze et al., 2015)
PTR-MS[1]	n.a.	*pptv*	0.1	molecular species other than the specific molecule in question	(Rieder et al., 2001, 2002; Summer et al., 2003)

REFERENCES

Accorsi, A., Morrone, B., Domenichini, I., Valenti, S., Raffi, G. B., & Violante, F. S. (2005). *Urinary sevoflurane and hexafluoro-isopropanol as biomarkers of low-level occupational exposure to sevoflurane.* https://doi.org/10.1007/s00420-004-0580-8

Accorsi, A., Valenti, S., Barbieri, A., Raffi, G. B., & Violante, F. S. (2003). Proposal for single and mixture biological exposure limits for sevoflurane and nitrous oxide at low occupational exposure levels. *International Archives of Occupational and Environmental Health*, *76*(2), 129–136. https://doi.org/10.1007/s00420-002-0379-4

Al-Ghanem, S., Battah, A. H., & Salhab, A. S. (2008). Monitoring of volatile anesthetics in operating room personnel using GC-MS. *Jordan Medical Journal*, *42*(1), 13–19.

Alessio, A., Zadra, P., Negri, S., Maestri, L., Imberti, R., Ghittori, S., Imbriani, M., & Cavalleri, A. (2003). *Monitoraggio biologico dell'esposizione occupazionale a Desflurane* (pp. 137–141).

Amiri, F., Neghab, M., Shouroki, F. K., Yousefinejad, S., & Hassanzadeh, J. (2018). Early, subclinical hematological changes associated with occupational exposure to high levels of nitrous oxide. *Toxics*, *6*(4), 4–13. https://doi.org/10.3390/toxics6040070

Ansalone, C. M., Sarcletti, G., Crespi, V., Cavallo, D. M., Borchini, R., & Ferreraio, M. (2004). *Messa a punto di un metodo di misura del sevoflurano urinario per il monitoraggio biologico della esposizione a basse concentrazioni.*

Barberio, C. J. C., Bolt, C. J. D., Austin, P. N., & Craig, M. W. J. (2006). Pollution of ambient air by volatile anesthetics: A comparison of 4 anesthetic management techniques. In *AANA Journal* (Vol. 74, Issue 2). www.aana.com/members/journal/

Barbic, F., Bagnati, M., Basile, M., Zanoli, M. V., Carettoni, L., Cassani, C., Porta, P., Fortina, A., Mantovani, C., & Bellomo, G. (2003). *L'esafluoroisopropanolo urinario nella valutazione dell'esposizione professionale a sevofluorano: Aspetti metodologici e punti critici.*

Blokker-Veldhuis, M. J., Rutten, P. M. M. J., & De Hert, S. G. (2011). Occupational exposure to sevoflurane during cardiopulmonary bypass. *Perfusion*, *26*(5), 383–389. https://doi.org/10.1177/0267659111409971

Braz, M. G., Carvalho, L. I. M., Chen, C. Y. O., Blumberg, J. B., Souza, K. M., Arruda, N. M., Filho, D. A. A., Resende, L. O., Faria, R. T. B. G., Canário, C. d. A., de Carvalho, L. R., Corrêa, C. R., Braz, J. R. C., & Braz, L. G. (2020). High concentrations of waste anesthetic gases induce genetic damage and inflammation in physicians exposed for three years: A cross-sectional study. *Indoor Air*, *30*(3), 512–520. https://doi.org/10.1111/ina.12643

Buratti, M., Pellegrino, O., Valla, C., & Colombi, A. (1993). *Monitoraggio biologico dell'esposizione professionale a gas e vapori anestetici: determinazione di protossido d'azoto, alotano e isoflurano dell'urina.*

Byhahn, C., Heller, K., Lischke, V., & Westphal, K. (2001). Surgeon's Occupational Exposure to Nitrous Oxide and Sevoflurane during Pediatric Surgery. *World J. Surg*, *25*, 1109–1112. https://doi.org/10.1007/s00268-001-0089-1

Byhahn, C., Wilke, H.-J., Strouhal, U., Kessler, P., & Westphal, K. (2000). *Occupational exposure to nitrous oxide and desflurane during ear-nose-throat-surgery.*

Cakmak, G., Eraydın, D., Berkkan, A., Yagar, S., & Burgaz, S. (2019). *Genetic damage of operating and recovery room personnel occupationally exposed to waste anaesthetic gases.* https://doi.org/10.1177/0960327118783532

Chaoul, M. M., Braz, J. R. C., Lucio, L. M. C., Golim, M. A., Braz, L. G., & Braz, M. G. (2015). Does occupational exposure to anesthetic gases lead to increase of pro-inflammatory cytokines?

Inflammation Research, *64*(12), 939–942. https://doi.org/10.1007/s00011-015-0881-2

Cheng, N.-Y. Y., Chuang, H.-C. C., Shie, R.-H. H., Liao, W.-H. H., & Hwang, Y.-H. H. (2019). Pilot Studies of VOC Exposure Profiles during Surgical Operations. *Annals of Work Exposures and Health*, *63*(2), 173–183. https://doi.org/10.1093/annweh/wxy099

Cheung, S. K., Özelsel, T., Rashiq, S., & Tsui, B. C. (2016). Postoperative environmental anesthetic vapour concentrations following removal of the airway device in the operating room versus the posthanesthesia care unit. *Canadian Journal of Anesthesia*, *63*(9), 1016–1021. https://doi.org/10.1007/s12630-016-0678-y

Coleman, M. A., Coles, S., Lytle, T., & Bennetts, F. E. (1994). *Prevention of atmospheric contamination during isoflurane sedation.*

Cope, K. A., Merritt, W. T., Krenzischek, D. A., Schaefer, J., Bukowski, J., Foster, W. M., Bernacki, E., Dorman, T., & Risby, T. H. (2002). Phase II collaborative pilot study: Preliminary analysis of central neural effects from exposure to volatile anesthetics in the PACU. *Journal of Perianesthesia Nursing*, *17*(4), 240–250. https://doi.org/10.1053/jpan.2002.34167

Dang, B., Esrryn-Behar, M., Maillard, M. F., Bourdon, R., & Saint-Maurice, C. (1992). *Theatre staff members and exposure to halogenated agents*. 279–284.

De-Amici, D., Ceriana, P., Lodola, L., Rolandi, L., & Zecca, E. (1996). *Caratteristiche qualitative dell'esposizione umana a contaminanti chimici aerodispersi in sale operatorie.*

Dehghani, F., Kamalinia, M., Omidi, F., & Fallahzadeh, R. A. (2021). Probabilistic health risk assessment of occupational exposure to isoflurane and sevoflurane in the operating room. *Ecotoxicology and Environmental Safety*, *207*(September 2020), 111270. https://doi.org/10.1016/j.ecoenv.2020.111270

Domenegati, E., Rolandi, L., Lodola, L., Bressan, M. A., Ceriana, P., & Filisetti, P. (1996). *Valutazione di un protocollo di sorveglianza sanitaria per il personale esposto ad anesttici inalatori in tre sale operatorie campione* (pp. 267–305).

Eroglu, A., Celep, F., & Erciyes, N. (2006). A comparison of sister chromatid exchanges in lymphocytes of anesthesiologists to nonanesthesiologist in the same hospital. *Anesthesia and Analgesia*, *102*(5), 1573–1577. https://doi.org/10.1213/01.ane.0000204298.42159.0e

Gentili, A., Accorsi, A., Pigna, A., Bachiocco, V., Domenichini, I., Baroncini, S., & Violante, F. S. (2004). *Exposure of personnel to sevoflurane during paediatric anaesthesia: influence of professional role and anesthetic procedure* (pp. 638–645).

Ghimenti, S., Tabucchi, S., Bellagambi, F. G., Lomonaco, T., Onor, M., Trivella, M. G., Fuoco, R., & Di Francesco, F. (2015). Determination of sevoflurane and isopropyl alcohol in exhaled breath by thermal desorption gas chromatography-mass spectrometry for exposure assessment of hospital staff. *Journal of Pharmaceutical and Biomedical Analysis*, *106*, 218–223. https://doi.org/10.1016/j.jpba.2014.11.052

Gianella, M., Hahnloser, D., Rey, J. M., & Sigrist, M. W. (2014). Quantitative chemical analysis of surgical smoke generated during laparoscopic surgery with a vessel-sealing device. *Surgical Innovation*, *21*(2), 170–179. https://doi.org/10.1177/1553350613492025

Gobbo Braz, L., Reinaldo, J., Braz, C., Aparecido, G., Cavalcante, S., Souza, K. M., Mendes De Carvalho Lucio, L., & Gobbo Braz, M. (2017). Comparison of waste anesthetic gases in operating rooms with or without an scavenging system in a Brazilian University Hospital. *Brazilian Journal of Anesthesiology (English Edition)*, *67*(5), 516–520. https://doi.org/10.1016/j.bjane.2017.04.008

González-Rodríguez, R., Muñoz Martínez, A., Galan Serrano, J., & Moral García, M. V. (2014). Health worker exposure risk during inhalation sedation with sevoflurane using the (AnaConDa®)

anaesthetic conserving device. *Revista Espanola de Anestesiologia y Reanimacion*, *61*(3), 133–139. https://doi.org/10.1016/j.redar.2013.11.011

Gustorff, B., Lorenzl, N., Aram, L., Krenn, C. G., Jobst, B. P., & Hoerauf, K. H. (2002). Environmental monitoring of sevoflurane and nitrous oxide using the cuffed oropharyngeal airway. *Anesthesia and Analgesia*, *94*(5), 1244–1248. https://doi.org/10.1097/00000539-200205000-00036

Hall, J. E., Henderson, K. A., Oldham, T. A., Pugh, S., & Harmer, M. (1997). Environmental monitoring during gaseous induction with sevoflurane. In *British Journal of Anaesthesia* (Vol. 79).

Haufroid, V., Cardinal, S., Licot, C., Villalpando, M. L., Van Obbergh, L., Clippe, A., & Lison, D. (2000). Biological monitoring of exposure to sevoflurane in operating room personnel by the measurement of hexafluoroisopropanol and fluoride in urine. *Biomarkers*, *5*(2), 141–151. https://doi.org/10.1080/135475000230451

Heiderich, S., Thoben, C., Dennhardt, N., Koppert, W., Krauß, T., Sümpelmann, R., Zimmermann, S., & Klingler, W. (2018). Low anaesthetic waste gas concentrations in postanaesthesia care unit. *European Journal of Anaesthesiology*, *35*(7), 534–538. https://doi.org/10.1097/EJA.0000000000000785

Heijbel, H., BjurstÖm, R., & Jakobsson, J. G. (2010). Personnel breathing zone sevoflurane concentration adherence to occupational exposure limits in conjunction with filling of vaporisers. *Acta Anaesthesiologica Scandinavica*, *54*(9), 1117–1120. https://doi.org/10.1111/j.1399-6576.2010.02280.x

Henderson, K. A., & Matthews, I. P. (1999). An environmental survey of compliance with occupational exposure standards (OES) for anaesthetic gases. *Anaesthesia*, *54*(10), 941–947. https://doi.org/10.1046/j.1365-2044.1999.01032.x

Henderson, K. A., & Matthews, I. P. (2000). Staff exposure to anaesthetic gases in theatre and non theatre areas. *European Journal of Anaesthesiology*, *17*(3), 149–151. https://doi.org/10.1046/j.1365-2346.2000.00620.x

Herzog-Niescery, J., Botteck, N. M., Vogelsang, H., Gude, P., Bartz, H., Weber, T. P., & Seipp, H. M. (2015). Occupational chronic sevoflurane exposure in the everyday reality of the anesthesia workplace. *Anesthesia and Analgesia*, *121*(6), 1519–1528. https://doi.org/10.1213/ANE.0000000000001015

Herzog-Niescery, J., Gude, P., Gahlen, F., Seipp, H.-M., Bartz, H., Botteck, N. M., Bellgardt, M., Dazert, S., Weber, T. P., & Vogelsang, H. (2016). Surgeons' exposure to sevoflurane during paediatric adenoidectomy: a comparison of three airway devices. *Anaesthesia*, *71*(8), 915–920. https://doi.org/10.1111/anae.13515

Herzog-Niescery, J., Seipp, H.-M., Bellgardt, M., Herzog, T., Belyaev, O., Waldemar, U., Gude, P., Weber, T., & Vogelsang, H. (2020). *Comparison of 3 Methods to Asses Occupational Sevoflurane Exposure in Abdominal Surgeons: A Single-Center Observational Pilot Study.*

Herzog-Niescery, J., Vogelsang, H., Bellgardt, M., Botteck, N. M., Seipp, H.-M. M., Bartz, H., Weber, T. P., & Gude, P. (2017). The child's behavior during inhalational induction and its impact on the anesthesiologist's sevoflurane exposure. *Paediatric Anaesthesia*, *27*(12), 1247–1252. https://doi.org/10.1111/pan.13269

Herzog-Niescery, J., Vogelsang, H., Bellgardt, M., Seipp, H.-M., Weber, T. P., & Gude, P. (2019). The Personnel's Sevoflurane Exposure in the Postanesthesia Care Unit Measured by Photoacoustic Gas Monitoring and Hexafluoroisopropanol Biomonitoring. *Journal of Perianesthesia Nursing*, *34*(3), 606–613. https://doi.org/10.1016/j.jopan.2018.10.005

Herzog-Niescery, J., Vogelsang, H., Gude, P., Seipp, H. M., Bartz, H., Uhl, W., Weber, T. P., & Bellgardt, M. (2018). The impact of the anesthetic conserving device on occupational exposure to isoflurane among intensive care healthcare professionals. *Minerva Anestesiologica*, *84*(1), 25–32.

https://doi.org/10.23736/S0375-9393.17.11770-0

Herzog-Niescery, J., Vogelsang, H., Gude, P., Seipp, H. M., Uhl, W., Weber, T. P., & Bellgardt, M. (2019). Environmental safety: Air pollution while using MIRUS™ for short-term sedation in the ICU. *Acta Anaesthesiologica Scandinavica*, *63*(1), 86–92. https://doi.org/10.1111/aas.13222

Hiller, K. N., Altamirano, A. V, Cai, C., Tran, S. F., & Williams, G. W. (2015). *Evaluation of Waste Anesthetic Gas in the Postanesthesia Care Unit within the Patient Breathing Zone.* https://doi.org/10.1155/2015/354184

Hobbhahn, J., Hoerauf, K., Wiesner, G., Schrögendorfer, K., & Taeger, K. (1998). Waste gas exposure during desflurane and isoflurane anaesthesia. *Acta Anaesthesiologica Scandinavica*, *42*(7), 864–867. https://doi.org/10.1111/j.1399-6576.1998.tb05335.x

Hoerauf, K. H., Funk, W., Harth, M., & Hobbhahn, J. (1997). Occupational exposure to sevoflurane, halothane and nitrous oxide during paediatric anaesthesia. Waste gas exposure during paediatric anaesthesia. *Anaesthesia*, *52*(3), 215–219. https://doi.org/10.1111/j.1365-2044.1997.070-az0061.x

Hoerauf, K. H., Harth, M., Wild, K., & Hobbhahn, J. (1997). Occupational exposure to desflurane and isoflurane during cardiopulmonary bypass: is the gas outlet of the membrane oxygenator an operating theatre pollution hazard? In *British Journal of Anaesthesia* (Vol. 78).

Hoerauf, K. H., Hartmann, T., Acilovic, S., Kopp, A., Wiesner, G., Gustorff, B., Jellinek, H., & Krafft, P. (2001). Waste gas exposure to sevoflurane and nitrous oxide during anaesthesia using the oesophageal-tracheal combitube small adult™. In *British Journal of Anaesthesia* (Vol. 86, Issue 1, pp. 124–126). https://doi.org/10.1093/bja/86.1.124

Hoerauf, K. H., Hartmann, T., Zavrski, A., Adel, S., Rg Burger, H.-J., Koinig, H., & Zimpfer, M. (1999). *Occupational exposure to sevoflurane during sedation of adult patients.*

Hoerauf, K. H., Koller, C., Jakob, W., Taeger, K., Hobbhahn, J., Hoerauf, K. H., Koller, C., Jakob, W., Taeger, K., & Hobbhahn, J. (1996). Isoflurane waste gas exposure during general anaesthesia: the laryngeal mask compared with tracheal intubation The treatment assignments (tracheal tube or LMA) were contained in sequen. In *British Journal of Anaesthesia* (Vol. 77).

Hoerauf, K. H., Koller, C., Taeger, K., & Hobbhahn, J. (1996). Occupational exposure to sevoflurane and nitrous oxide in operating room personnel. *International Archives of Occupational and Environmental Health*, *69*(2), 134–138. https://doi.org/10.1007/s004200050127

Hoerauf, K. H., Wallner, T., Akça, O., Taslimi, R., & Sessler, D. I. (1999). Exposure to sevoflurane and nitrous oxide during four different methods of anesthetic induction. *Anesthesia and Analgesia*, *88*(4), 925–929. https://doi.org/10.1097/00000539-199904000-00045

Hua, H. X., Deng, H. B., Huang, X. L., Ma, C. Q., Xu, P., Cai, Y. H., & Wang, H. T. (2021). Effects of Occupational Exposure to Waste Anesthetic Gas on Oxidative Stress and DNA Damage. *Oxidative Medicine and Cellular Longevity*, *2021*. https://doi.org/10.1155/2021/8831535

IFA. (2015). *GESTIS International Limit Values GESTIS International Limit Values*. 2–3.

Imberti, R., Preseglio, I., Imbriani, M., Ghittori, S., Cimino, F., & Mapelli, A. (1995). Low flow anaesthesia reduces occupational exposure to inhalation anaesthetics Environmental and biological measurements in operating room personnel. *Acta Anaesthesiologica Scandinavica*, *39*(5), 586–591. https://doi.org/10.1111/j.1399-6576.1995.tb04132.x

Imbriani, M., & Ghittori, S. (1988). Evaluation of exposure to isoflurane (Forane): Environmental and biological measurements in operating room personnel. *Journal of Toxicology and Environmental Health*, *25*(4), 393–402. https://doi.org/10.1080/15287398809531219

Imbriani, M., Ghittori, S., & Pezzagno, G. (1998). *The Biological Monitoring of Inhalation Anaesthetics.*

Imbriani, M., Ghittori, S., Pezzagno, G., & Capodaglio, E. (1995). Anesthetic in urine as biological index of exposure in operating-room personnel. *Journal of Toxicology and Environmental Health*, *46*(2), 249–260. https://doi.org/10.1080/15287399509532032

Imbriani, M., Zadra, P., Negri, S., Alessio, A., Maestri, L., & Ghittori, S. (2001). *Monitoraggio biologico dell'esposizione professionale a sevoflurane.*

Jafari, A., Bargeshadi, R., Jafari, · Fatemeh, Mohebbi, I., Hajaghazadeh, · Mohammad, Jafari, F., Mohebbi, I., & Hajaghazadeh, M. (2018). Environmental and biological measurements of isoflurane and sevoflurane in operating room personnel. *International Archives of Occupational and Environmental Health*, *91*(3), 349–359. https://doi.org/10.1007/s00420-017-1287-y

Jafari, A., Jafari, F., & Mohebbi, I. (2020). Effects of occupational exposure to trace levels of halogenated anesthetics on the liver, kidney, and oxidative stress parameters in operating room personnel. *Toxin Reviews*, *39*(3), 242–251. https://doi.org/10.1080/15569543.2018.1498898

Jan-Peter Özelsel, T., Kim, S., Buro, K., & Tsui, B. (2018). Paediatric Post-Anaesthesia Care Unit. *Turk J Anaesthesiol Reanim*, *46*(5), 362–368. https://doi.org/10.5152/TJAR.2018.35683

Jankowska, A., Czerczak, S., Kucharska, M., Wesołowski, W., Maciaszek, P., & Kupczewska-Dobecka, M. (2015). Application of predictive models for estimation of health care workers exposure to sevoflurane. *International Journal of Occupational Safety and Ergonomics*, *21*(4), 471–479. https://doi.org/10.1080/10803548.2015.1086183

Ji, Z., Wu, W., Zhou, F., Hu, J., Xu, Q., Yang, W., Peng, X., Wang, X., Zhang, C., & Li, L. (2021). Effects of sevoflurane exposure on apoptosis and cell cycle of peripheral blood lymphocytes, and immunologic function. *BMC Anesthesiology*, *21*(1), 87. https://doi.org/10.1186/s12871-021-01305-w

Kampan, S. (2019). Air quality and employee hygiene-related behavior in a post anesthesia care unit in Thailand. *Asian/Pacific Island Nursing Journal*, *4*(1), 11–19. https://doi.org/10.31372/20190401.1005

Kunze, N., Weigel, C., Vautz, W., Schwerdtfeger, K., Jünger, M., Quintel, M., & Perl, T. (2015). Multi-capillary column-ion mobility spectrometry (MCC-IMS) as a new method for the quantification of occupational exposure to sevoflurane in anaesthesia workplaces: an observational feasibility study. *Journal of Occupational Medicine and Toxicology*, *10*(1), 1–9. https://doi.org/10.1186/s12995-015-0056-7

Li, S.-H., Li, S.-N., Shih, H.-Y., Chiou, S.-F., & Yi, H.-D. (2004). A local scavenging system to remove waste anesthetic gases during general anesthesia. *Acta Anaesthesiologica Taiwanica : Official Journal of the Taiwan Society of Anesthesiologists*, *42*(2), 61–67. http://www.ncbi.nlm.nih.gov/pubmed/15346700

Li, S.-H., Li, S.-N., Shih, H.-Y., Yi, H.-D., & Chiang, C.-Y. (2002). *Personnel Exposure to Waste Sevoflurane and Nitrous Oxide during General Anesthesia with Cuffed Endotracheal Tube.*

Lucchini, R., Belotti, L., Cassitto, M. G., Faillace, A., Margonari, M., Micheloni, G., Scapellato, M. L., Somenzi, V., Spada, T., Toffoletto, E., & Gilioli, R. (1997). *Neruobehavioral functions in operating theatre personnel: a multicenter study.*

Lucchini, R., Toffoletto, F., Camerino, D., Fazioli, R., Ghittori, S., Gilioli, R., Signorini, A., & Alessio, L. (1995). Neurobehavioral functions in operating theatre personnel exposed to anesthetic gases. *Medicina Del Lavoro*, *86*(1), 27–33.

Marracini, P., Vittadini, G., Ghittori, S., Giorgi, I., Bonelli, S., Buonocore, M., & Imbriani, M. (1992). *Valutazione di alcuni parametri neuropsicologici in soggetti professionalmente esposti ad anestetici.*

Mcglothlin, J. D., Moenning, J. E., & Cole, S. S. (2014). *Evaluation and Control of Waste Anesthetic Gases in the Postanesthesia Care Unit.* https://doi.org/10.1016/j.jopan.2013.09.010

Mierdl, S., Byhahn, C., Abdel-Rahman, U., Matheis, G., & Westphal, K. (2003). Occupational exposure to inhalational anesthetics during cardiac surgery on cardiopulmonary bypass. *Annals of Thoracic Surgery*, *75*(6), 1924–1927. https://doi.org/10.1016/S0003-4975(03)00003-1

Neghab, M., Kargar-Shouroki, F., Mozdarani, H., Yousefinejad, S., Alipour, H., & Fardid, R. (2020). Association between genotoxic properties of inhalation anesthetics and oxidative stress biomarkers. In *Toxicology and Industrial Health* (Vol. 36, Issue 6, pp. 454–466). https://doi.org/10.1177/0748233720935696

Norton, P., Pinho, P., Xará, D., Pina, F., & Norton, M. (2020). Assessment of anesthetic gases in a central hospital. *Porto Biomedical Journal*, *5*(4), e076. https://doi.org/10.1097/j.pbj.0000000000000076

Özelsel, T. J. P., Kim, S., Buro, K., & Tsui, B. (2018). Elevated waste anaesthetic gas concentration in the paediatric postanaesthesia care unit. *Turk Anesteziyoloji ve Reanimasyon Dernegi Dergisi*, *46*(5), 362–366. https://doi.org/10.5152/TJAR.2018.35683

Pickworth, T., Jerath, A., Devine, R., Kherani, N., Marcin, R. •, & ˛sowicz, W. (2013). *The scavenging of volatile anesthetic agents in the cardiovascular intensive care unit environment: a technical report La récupération des agents anesthésiques volatils dans l'environnement de l'unité des soins intensifs cardiovasculaires: un rapport tech.* https://doi.org/10.1007/s12630-012-9814-5

Prado, C., Tortosa, J. A., Ibarra, I., Luna, A., & Periago, J. F. (1997). Biological monitoring of occupational exposure to isoflurane by measurement of isoflurane exhaled breath. In *Journal of Applied Toxicology* (Vol. 17, Issue 3, pp. 179–183). https://doi.org/10.1002/(SICI)1099-1263(199705)17:3<179::AID-JAT425>3.0.CO;2-Y

Proietti, L., Duscio, D., Rotiroti, G., & Sandonà, P. B. (2000). *Monitoraggio ambientale e sorveglianza sanitaria per il personale esposto ad anestetici inalatori.*

Proietti, L., Longo, B., Giulino, S., & DUscio, D. (2003). *Tecniche di somministrazione di agenti anestetici per inalazione, esposizione professionale ed effetti neurocomportamentali precoci.*

Rahe-Meyer, N., Solomon, C., Vial, S., Gruber, M., Weilbach, C., Piepenbrock, S., & Winterhalter, M. (2009). Comparison of breathing tube connectors during invasive bronchial procedures. *Anaesthesia*, *64*(6), 666–673. https://doi.org/10.1111/j.1365-2044.2009.05894.x

Raj, N., Henderson, K. A., Hall, J. E., Aguilera, I. M., Harmer, M., Hutchings, A., & Williams, B. (2003). *Evaluation of personal, environmental and biological exposure of paediatric anaesthetists to nitrous oxide and sevoflurane*.*

Ravagli, A., Spalla, B., Marraccini, P., & Franco, G. (1987). Anesthetics vapors and gases and health hazards: results of an environmental survey. *Giornale Italiano Di Medicina Del Lavoro*, *9*(1), 39–44.

Rieder, J., Keller, C., Brimacombe, J., Gruber, G., Lirk, P., Summer, G., & Amann, A. (2002). *Monitoring pollution by proton-transfer-reaction mass spectrometry during paediatric anaesthesia with positive pressure ventilation via the laryngeal mask airway or uncuffed tracheal tube.*

Rieder, J., Prazeller, P., Boehler, M., Lirk, P., Lindinger, W., & Amann, A. (2001). Online monitoring of air quality at the postanesthetic care unit by proton-transfer-reaction mass spectrometry. *Anesthesia and Analgesia*, *92*(2), 389–392. https://doi.org/10.1213/00000539-200102000-00021

Ritzu, S., Boccalon, P., Sanchez, M. A., Arcangeli, G., & Capelli, V. (2007). *ESPOSIZIONE A GAS ANESTETICI: RISULTATI DI 13 ANNI DI MONITORAGGIO AMBIENTALE E BIOLOGICO IN UN' AZIENDA OSPEDALIERA.*

Rovesti, S., Ferrari, A., Faggiano, D., & Vivoli, G. (2005). *Monitorggio dell'esposizione professionale ad anestetici volatili in sala operatoria: misurazioni ambientali e biologiche.*

Sackey, P. V., Martling, C. R., Nise, G., & Radell, P. J. (2005). Ambient isoflurane pollution and isoflurane

consumption during intensive care unit sedation with the Anesthetic Conserving Device. *Critical Care Medicine*, *33*(3), 585–590. https://doi.org/10.1097/01.CCM.0000156294.92415.E2

Sárkány, P., Tankó, B., Simon, É., Gál, J., Fülesdi, B., & Molnár, C. (2016). Does standing or sitting position of the anesthesiologist in the operating theatre influence sevoflurane exposure during craniotomies? *BMC Anesthesiology*, *16*(1), 1–5. https://doi.org/10.1186/s12871-016-0284-0

Scapellato, M. L., Carrieri, M., Maccà, I., Salamon, F., Trevisan, A., Manno, M., & Bartolucci, G. B. (2014). Biomonitoring occupational sevoflurane exposure at low levels by urinary sevoflurane and hexafluoroisopropanol. *Toxicology Letters*, *231*(2), 154–160. https://doi.org/10.1016/j.toxlet.2014.10.018

Schebesta, K., Lorenz, V., Schebesta, E. M., HÖrauf, K., Gruber, M., Kimberger, O., Chiari, A., Frass, M., & Krafft, P. (2010). Exposure to anaesthetic trace gases during general anaesthesia: CobraPLA vs. LMA classic. *Acta Anaesthesiologica Scandinavica*, *54*(7), 848–854. https://doi.org/10.1111/j.1399-6576.2009.02194.x

Sessler, D. I., & Badgwell, J. M. (1998). Exposure of postoperative nurses to exhaled anesthetic gases. *Anesthesia and Analgesia*, *87*(5), 1083–1088. https://doi.org/10.1097/00000539-199811000-00019

Sitarek, K., Werolowski, W., Kucharska, M., & Celichowski, G. (2000). *CONCENTRATIONS OF ANAESTHETIC GASES IN HOSPITAL OPERATING THEATRES*.

Souza, K. M., Braz, L. G., Nogueira, F. R., Souza, M. B., Bincoleto, L. F., Aun, A. G., Corrente, J. E., Carvalho, L. R., Braz, J. R. C., & Braz, M. G. (2016). Occupational exposure to anesthetics leads to genomic instability, cytotoxicity and proliferative changes. *Mutation Research/Fundamental and Molecular Mechanisms of Mutagenesis*, *791–792*, 42–48. https://doi.org/10.1016/j.mrfmmm.2016.09.002

Summer, G., Lirk, P., Hoerauf, K., Riccabona, U., Bodrogi, F., Raifer, H., Deibl, M., Rieder, J., & Schobersberger, W. (2003). Sevoflurane in exhaled air of operating room personnel. *Anesthesia and Analgesia*, *97*(4), 1070–1073. https://doi.org/10.1213/01.ANE.0000081796.67539.27

Tankó, B., Molnár, C., Budi, T., Peto, C., Novák, L., & Fülesdi, B. (2009). The relative exposure of the operating room staff to sevoflurane during intracerebral surgery. *Anesthesia and Analgesia*, *109*(4), 1187–1192. https://doi.org/10.1213/ane.0b013e3181b0cbea

Tanser, S. J., & Johnson, A. (2002). Evaluation of a new paediatric scavenging valve. *Paediatric Anaesthesia*, *12*(5), 448–450. https://doi.org/10.1046/j.1460-9592.2002.00835.x

Virgili, A., Scapellato, M. L., Maccà, I., Perini, M., Carrieri, M., Gori, G., Saia, B., & Bartolucci, G. B. (2002). *Esposizione professionale a gas anestetici in alcuni ospeddlai del Veneto* (pp. 447–450).

Wiesner, G., Harth, M., Hoerauf, K., Szulc, R., Jurczyk, W., Sobczynski, P., Hobbhahn, J., & Taeger, K. (2000). Occupational exposure to inhaled anaesthetics: A follow-up study on anaesthetists of an eastern European university hospital. *International Archives of Occupational and Environmental Health*, *74*(1), 16–20. https://doi.org/10.1007/s004200000189

Wiesner, G., Harth, M., Szulc, R., Jurczyk, W., Sobczynski, P., Hoerauf, K. H., Hobbhahn, J., & Taeger, K. (2001). A follow-up study on occupational exposure to inhaled anaesthetics in Eastern European surgeons and circulating nurses. *International Archives of Occupational and Environmental Health*, *74*(1), 16–20. https://doi.org/10.1007/s004200000189

Williams, G. W., Gumbert, S. D., Pivalizza, E. G., Syed, T. A., Burnett, T., Mancillas, O. L., Vargas, L. A., Ahn, S. H., Cai, C., & Hagberg, C. A. (2019). *Evaluation and control of waste anesthetic gas in the postanesthesia care unit within patient and caregiver breathing zones*. https://doi.org/10.1080/08998280.2018.1502017

Zaffina, S., Camisa, V., Poscia, A., Tucci, M. G., Montaldi, V., Cerabona, V., Wahocka, M., & Moscato, U. (2012). *Esposizione professionale al Sevoflurano nelle sale operatorie di pertinenza pediatrica: il contributo del monitoraggio ambientale con tecnica multi-punto nella valutazione del rischio.*

Zanetti, C., Fiorio, S., Moretto, A., Foresto, F., Baggio, R., Gardin, F., & Scarpazza, F. (2004). *Studio longitudinale (16 anni) della salute riproduttiva di 61 lavoratrici esposte a livelli misurati di anestetici volatili.*

Zare Sakhvidi, M. J., Barkhordari, A., Salehi, M., Behdad, S., & Fallahzadeh, H. (2013). Application of mathematical models in combination with monte carlo simulation for prediction of isoflurane concentration in an operation room theater. *Industrial Health*, *51*(5), 545–551. https://doi.org/10.2486/indhealth.2012-0130
